# Refining the Genomic Region Containing a Major Locus Controlling Fruit Maturity in Peach

**DOI:** 10.1038/s41598-019-44042-4

**Published:** 2019-05-17

**Authors:** H. Elsadr, S. Sherif, T. Banks, D. Somers, S. Jayasankar

**Affiliations:** 1 University of Guelph, Department of Plant Agriculture, Ontario, Canada; 2 Alson H. Smith Jr. Agricultural Research and Extension Center, Virginia Tech, Winchester, Virginia USA; 3 Vineland Research and Innovation Centre, Department of Plant Agriculture, University of Guelph, Guelph, Ontario Canada

**Keywords:** Genetics, Plant breeding

## Abstract

Maturity date (MD), defined as the duration between the first calendar day of the year and maturity, and fruit development period (FDP), defined as the duration between full bloom and maturity, are highly variable in peach [*Prunus persica* (L.) Batsch]. There is a need to discover molecular markers associated with these traits in order to enhance the efficiency and reliability of breeding for extending the harvest season in peach. An association mapping population consisting of 132 peach accessions was phenotypically evaluated for MD and FDP, and genotypically characterized using the genotyping-by-sequencing (GBS) approach. The phenotypic and genotypic data collected were used to conduct a genome-wide association study (GWAS). The GWAS identified three SNPs on chromosome 4 that are significantly associated with both FDP and MD. These three SNPs covered a region of 43,067 bp; we referred to this region as the MD/FDP locus. Seven genes were identified in the MD/FDP locus. One or more of these genes is believed to regulate some aspect of maturity in peach. The data reported here is expected to aid in marker-assisted seedling selection (MASS) targeted towards widening peach germplasm for maturity, particularly early maturity.

## Introduction

The majority of traits selected for breeding programs in peach are related to productivity, growth habit, canopy structure, disease resistance, and fruit size, shape, color, texture, pubescence and sugar content^1^. More recently, several other aspects of the peach tree and fruit characteristics including expanding environmental ranges, reducing chilling requirements, increasing frost tolerances, enhancing fruit quality and appearance, improving shelf life and expanding the harvest season^2^ have become important traits targeted by peach breeders.

Fruit maturity is highly variable in peach [(*Prunus persica* (L.) Batsch]. The genetics of peach fruit maturity has only recently been studied and very little is understood regarding this important marketing trait. Maturity date (MD) (syn; ripening date; harvest date) in peach is defined as the duration of time, usually expressed in Julian days, between the first calendar day of the year and the harvest date^3^. The harvest date is defined as the calendar or Julian day when a certain percentage of peaches, as defined by the study, have attained maturity. Fruit development period (FDP) (syn; date to maturity, days to maturity; fruit ripening season) in peach is defined as the duration of time, expressed in days, between full bloom and harvest date. FDP in peach can range between two to nine months^4^. MD and FDP tend to be highly correlated since peaches that take longer to mature from flowering (FDP) also take longer to mature from the first Julian day of the year (MD). Peaches have three main MD/FDP phenotypes: (1) early maturing/short FDP, (2) mid-maturing/mid FDP and (3) late maturing/long FDP. Minor variability exists within each class of MD/FDP.

The wide range of MD/FDP found within both wild and commercial peach genotypes is desirable to producers, marketers and consumers. The FDP of wild peaches typically ranges from medium to late maturing (~120–210 days); it is rare to find wild type early maturing peaches^4^. Commercial peaches have a much larger FDP range (55–270 days) and include many early and very late maturing classes compared to their wild type counterparts^4^. Bassi *et al*. classified early, mid and late-maturing peach cultivars as those genotypes that have an FDP up to 90, 91–125 and over 125 days, respectively^5^. There is an interest among peach growers to widen the range of MD/FDP to accommodate fresh peach markets and spread out market risks and production costs^6–9^. Variations in MD/FDP may be utilized by producers to lengthen the harvest season, thereby, reducing sudden labour demands and costs while extending the market season. This practice will (1) lower costs and reduce losses of fruit due to post harvest diseases in storage, (2) increase producer income by spreading out sales, (3) allow an expansion of sales into new markets and (4) permit an increase in local production and consumption. In turn, peach consumers will enjoy a fresher more appealing peach for an extended season.

The heritability of MD/FDP has been evaluated in several studies and found to be relatively high^10–13^. de Souza *et al*., reported that neither MD or FDP had permanent environmental and/or nonadditive genetic effects (h2 = repeatability)^13^. Relatively high heritability estimates, coupled with high standard deviation values reported for these traits, indicate that selection for MD/FDP towards earliness or lateness should be quite effective. It is also suggested that breeding advances associated with increasing MD/FDP in peach are expected to be relatively rapid^13^. Depending on the population, one generation of independent selection for early ripening and short FDP genotypes would result in a 22–32% (31–35 day) decrease in ripening time^13^. By contrast, one generation of selection for late ripening and long FDP genotypes would result in a 40–90% (64–93 day) increase in maturity time^13^. Based on these results, selection for lateness would be approximately three times more effective for long FDP than for short FDP^13^. It is still not clearly evident whether MD/FDP is under additive polygenetic control or if a few genes with relatively large effects control this trait.

According to some studies, the trait regulating MD/FDP is quantitatively inherited and regulated by major genes. The idea that major genes act to regulate MD/FDP arises from two main observations. The first observation is that maturity measurements in large progenies, from parents that are distant in terms of MD/FDP, exhibit a bimodal or trimodal distribution. Furthermore, the MD/FDP of all of the offspring from these parents either mature within the parental MD/FDP dates or later/earlier than the parents^5^. The second observation is that bud sports, mutants from commercial cultivars, show FDP that are approximately separated by weekly intervals^4^. Bassi *et al*. used six progenies from controlled crosses of early, mid and late maturing cultivars to study the heritability of MD/FDP^5^. The group reported a mix of normal, uni-, bi- and tri-modal distribution frequency patterns for this trait. Crosses between mid-maturing x early-maturing cultivars produced offspring that tended towards lateness. Crosses between early-maturing x late-maturing cultivars produced offspring that tended towards lateness^5^. Such different patterns of frequency distribution for MD/FDP observed by Bassi *et al*. suggested that a few oligogenes and some minor genes with an additive effect act together to regulate this trait^5^. Furthermore, the mixed frequency distribution patterns observed for MD/FDP indicate that the cross combination ability between parents used for a cross plays an important role in determining whether progeny will lean towards earliness or lateness^5,14^.

By contrast, other studies have reported that the MD/FDP trait is a quantitative character under the control of QTLs^15–17^. Cantín *et al*. found that MD/FDP demonstrated a normal distribution, which indicates that these traits are inherited in a quantitative manner^7^. Recently, a fine mapping approach using two different segregating F2 populations has examined the genetic control of MD in peach^18^.

The efforts put into genomic information and tools have and will continue to be useful for crop improvement. Currently, transgenic manipulation of the agronomically important traits in peach is inefficient; however, some success has been attained^4,19,20^. The use of genomic data for marker assisted seedling selection (MASS) in peach has many applications to crop improvement, as it will allow breeders to cull undesirable progeny from crosses shortly after germination, and hence, reduce the time, expense and effort of maintaining and evaluating large numbers of progeny^2^. MASS will be particularly useful for assessing mature reproductive traits, such as flowering, fruiting and chilling requirements, as these traits can only be evaluated after several years of tree growth^2^. There is still controversy regarding the complete genetic control of early, mid and late maturing peaches. To date, genetic markers have been identified, which explain a percentage of the MD/FDP trait, however, the gene(s) controlling MD/FDP has yet to be determined. Epistatic interactions between a few major genes under the influence of minor genes or QTLs are believed to be responsible for the degree of variability observed in crosses made between early, mid and late maturing phenotypes.

There were three main objectives associated with this research. The objectives of the current study were to (1) examine the variability of MD and FDP that lies within several genotypes of peach, (2) identify SNP markers and genomic regions that associated with the traits evaluated, (3) investigate the putative functions, based on searches for orthologs, of some of the genes that co-located with the identified SNPs or within the genomic regions identified by the GWAS. GWAS differed from other studies on MD/FDP, which focused on bi-parental mapping populations and therefore determined loci that were specific to an explicit population and not necessarily to the genetic makeup of the species in general. Our hypotheses were (1) peach genotypes within our population would be highly variable in MD and FDP, (2) genomic regions regulating MD and FDP would be identified, (3) genes or gene orthologs with functions associated with MD and FDP in peach or other crops would be identified in the genomic regions that associated with maturity.

## Results and Discussion

### Phenotypic evaluation of the MD/FDP trait

The fruit tree breeding program at the University of Guelph has over 200 named cultivars and breeding lines of peaches that range from early to late maturing expressing both major and minor MD/FDP phenotype^3^. These genotypes are also grown in several other localities within Canada and the United States of America (USA), and therefore, an assessment of Genotype x Environment (GxE) interactions and their influence on MD/FDP is also conceivable. Therefore, this study is the first to detail the markers associated with MD/FDP over a wide range of genotypes from genetically distinct peach accessions over a range of environments.

In order to fulfill our objectives and address our hypotheses, trees from 132 genetically distinct peach genotypes from the breeding program were phenotyped for MD and FDP (Table S1). Qualitative and quantitative assessment of MD/FDP has been described in several studies^4,9,17,21^. In order to measure MD/FDP, certain parameters had to be decided to determine what defines flowering and what defines a mature fruit. Flower development in peach is dependent on temperature; with warmer temperatures accelerating floral expansion and opening. Spring floral development occurs in stages from the dormant bud stage to the full bloom stage. Fertilized fruit then continue to grow until they reach maturity. Usually, fruit destined for markets are harvested when they are “market mature”. Market maturity differs from physiological and ripened maturity. Market mature fruit are usually harvested shortly after physiological maturity when their flesh is still hard and when they have reached acceptable colour development as defined by the market and the cultivar grown. For the purpose of this study, a more accurate assessment of MD/FDP had to be considered to take into account environmental variability and variability associated with minor MDs/FDPs within major MDs/FDPs. Each accession was phenotypically evaluated for (i) bloom date (BD) (Julian day when ~50% of the flowers on the tree had reached the full bloom stage) and (ii) Maturity date (MD) (Julian day when ~50% of the fruit on the tree had reached the market mature stage). FDP was then calculated as the difference between BD and MD.

The yearly and mean minimum and maximum values, standard deviations (SD) and means of MD and FDP were generated (Table 1). Mean FDPs in 2012 were one week longer than 2013 due to variations in climatic conditions observed between these two years. In 2012, conditions were cooler and wetter during most of the growing season; while in 2013, conditions were warmer and dryer; therefore, fruit took longer to mature in 2012. Despite the longer FDPs observed in 2012 compared to 2013, mean MD values in 2012 were 10 days shorter compared to mean MD values in 2013. Therefore, the shorter MD values observed in 2012 are likely attributed to the earlier onset of flowering in 2012 and not due to reduced FDPs.

**Table 1:** Broad sense heritability, correlation coefficient, mean, minimum and maximum values, and standard deviation (SD) of maturity date and fruit development period in the VRIC association mapping population for two successive years.

Trait	Years	H^2^	Correlation	Mean	Min	Max	SD
MD	2012/2013/mean	0.11	0.98	223/233/228	190/198/194	263/273/272	18.6/17.5/18.5
FDP	2012/2013/mean	0.09	0.98	115/108/112	82/74/78	158/150/154	18.9/17.9/18.4

H^2^, broad sense heritability; SD, standard deviation.

Maturity date is expressed in Julian days at harvest.

Yearly phenotypic correlations for MD and FDP were used to generate Pearson year to year correlations (Table 1). Highly significant (P < 0.01) positive correlations were observed between 2012/2013 MD values (r_p_ = 0.98) and 2012/2013 FDP values (r_p_ = 0.98). Previous studies have also reported relatively strong (*r* > 0.90) year to year correlations for MD and FDP^10^. These results indicate that correlations between MD and FDP are stable across years.

Average phenotypic and genotypic data from MD and FDP were used to generate Pearson trait to trait phenotypic and genotypic correlations. Significant positive phenotypic correlations (r = 0.99, P < 0.01) and genotypic correlations (r = 1.00, P < 0.01) were observed between MD and FDP. Such positive correlations between MD and FDP has been previously reported^13^. The strong positive correlations between MD and FDP indicated that selection for early maturity will almost always result in selections that develop their fruit over a shorter duration of time from flowering; MD and FDP are reliable predictors of one another.

### Heritability of MD and FDP

The broad sense heritability of MD and FDP were estimated to be 0.09 and 0.11, respectively (Table 1). In previous studies the heritability of MD/FDP has been observed to range between 0.67 and 0.99, with most estimates being above 0.80^5,10,11,13,16,21^. The broad sense heritability values observed in this study are significantly lower compared to estimates reported in previous studies. This could be due to the difference between MDs/FDPs for each genotype observed across years, or the fact that previous studies used bi-parental mapping populations to calculate H^2^ while in this study an association panel consisting of a diverse and unrelated population was used to estimate H^2^ ^9,20^. Year to year differences in MD/FDP between genotypes likely resulted from differences in climatic conditions between the 2012 and 2013 growing seasons. The high variation explained by the genotype x year effect compared to genotype effect alone observed for both MD and FDP (Table 2) supports the hypothesis that the low H^2^ estimates observed in this study may at least be partially a result of variability in climatic conditions between the 2012 and 2013 growing seasons. Furthermore, a large residual effect was observed for MD and FDP (Table 2). This effect may be a result of the lack of partitioning of the variance components into tree (field replicate) and genotype x tree effect since trees were evaluated as a single unit.

**Table 2:** Variance components and standard deviations of the variance components for the maturity date and fruit development period traits evaluated over 2 years in the study population.

Trait	Genotype	SD	Year	SD	Genotype: Year	SD	Residual	SD
MD	19.91	4.46	8.70	2.95	49.82	7.06	278.83	16.70
FDP	17.66	4.20	0.00	0.00	45.46	6.74	294.26	17.15

SD, standard deviation.

### Frequency distribution of MD and FDP

**Figure 1:**
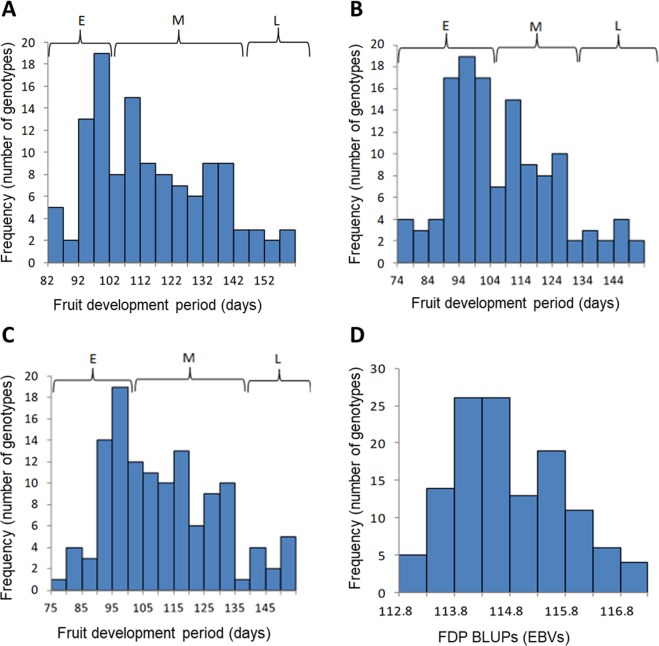
Frequency distribution of the peach FDP trait in the association mapping population. (**A**) Histogram of 2012 FDP phenotypic data, (**B**) Histogram of 2013 FDP phenotypic data, (**C**) Histogram of averaged FDP phenotypic data, (**D**) Histogram of FDP best linear unbiased predictors (BLUPs) expressed as estimated breeding values (EBVs). E, M and L refer to early, mid and late-maturing, respectively.

**Figure 2:**
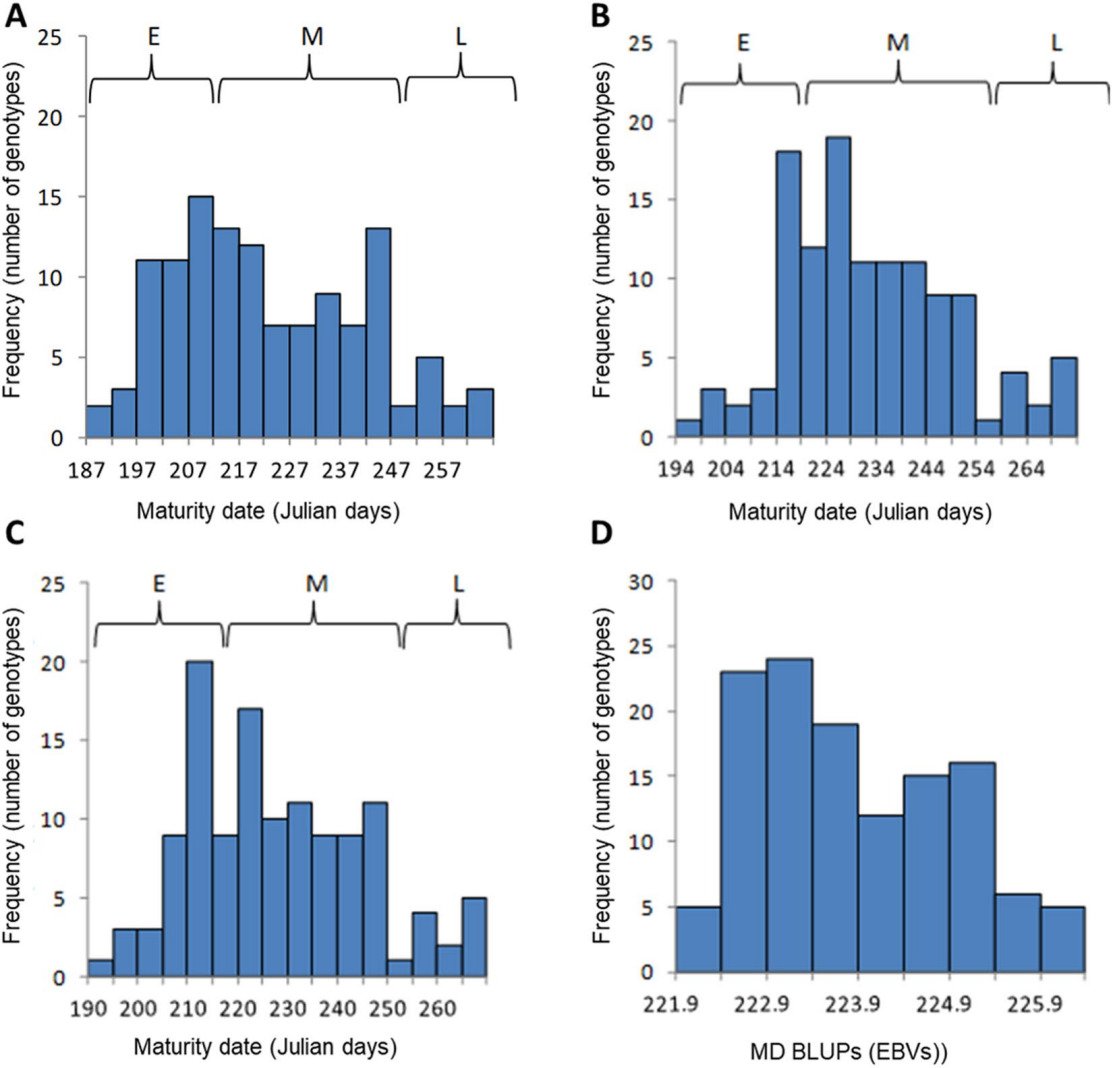
Frequency distribution of the peach MD trait in the association mapping population. (**A**) Histogram of 2012 MD phenotypic data, (**B**) Histogram of 2013 MD phenotypic data, (**C**) Histogram of averaged MD phenotypic data, (**D**) Histogram of MD best linear unbiased predictors (BLUPs) expressed as estimated breeding values (EBVs). E, M and L refer to early, mid and late-maturing, respectively.

The MD and FDP BLUPs and yearly phenotypic values and mean values did not have a normal frequency distribution (Figs 1 and 2d). A review of the literature indicated that MD and FDP typically have a bimodal or trimodal distribution in most populations^18,22,23^. A bimodal distribution usually indicates that two main MD/FDP phenotypes exist in a population; namely early and mid-maturing, mid and late-maturing or early and late-maturing phenotypes. A trimodal distribution usually indicates that three main MD/FDP phenotypes exist in a population; namely early, mid and late-maturing phenotypes. We categorized our MD/FDP data into bins of five day intervals to identify variations in modality that may be associated with these traits. The shape of our frequency distribution resembled the tri-modal distribution reported by Pirona *et al*.^18^. Furthermore, we observed three main haplotypes in our population that associated with the MD/FDP phenotypic data. These haplotypes corresponded to early, mid and late maturity. Based on this information we suggest that our yearly and mean MD/FDP frequency distributions were closer to trimodality than bimodality (Figs 1 and 2A–C) following a Mendelian behaviour of inheritance as has been reported in previous studies^5^.

### SNP discovery for fruit development period and maturity date

**Figure 3:**
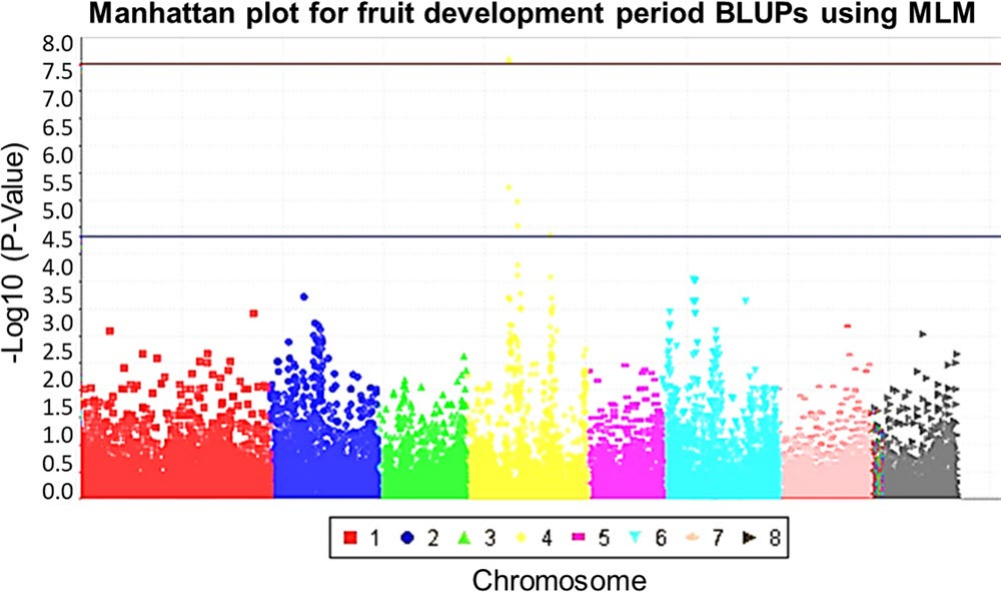
Manhattan plot for fruit development period (FDP) best linear unbiased predictors (BLUPs) using a mixed linear model (MLM). Red and blue lines represent significance thresholds at P < 0.01 and P < 0.05, respectively, using a Benjamini-Hochberg false discovery rate (BH FDR) adjustment.

**Figure 4:**
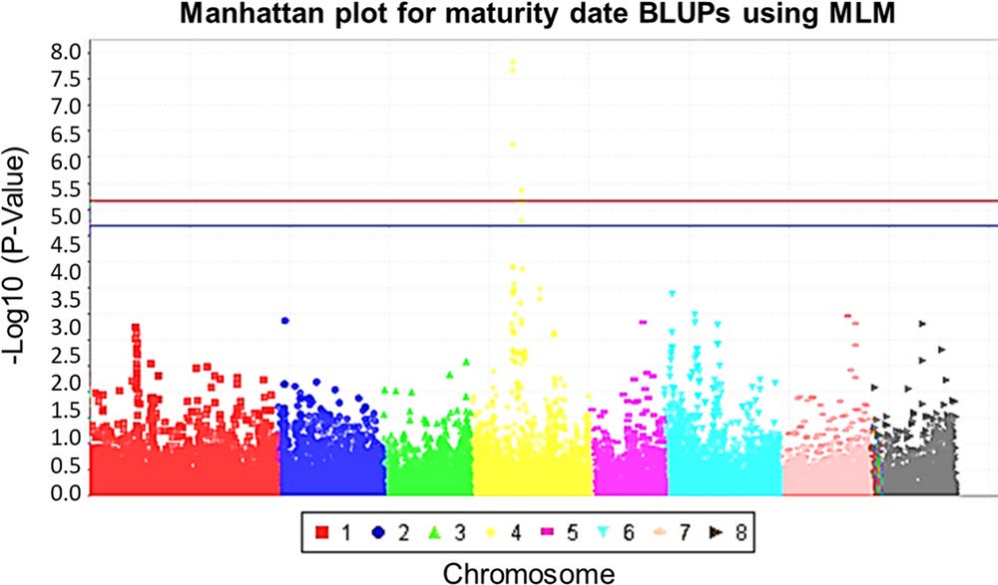
Manhattan plot for maturity date (MD) best linear unbiased predictors (BLUPs) using a mixed linear model (MLM). Red and blue lines represent significance thresholds at P < 0.01 and P < 0.05, respectively, using a Benjamini-Hochberg false discovery rate (BH FDR) adjustment.

**Figure 5:**
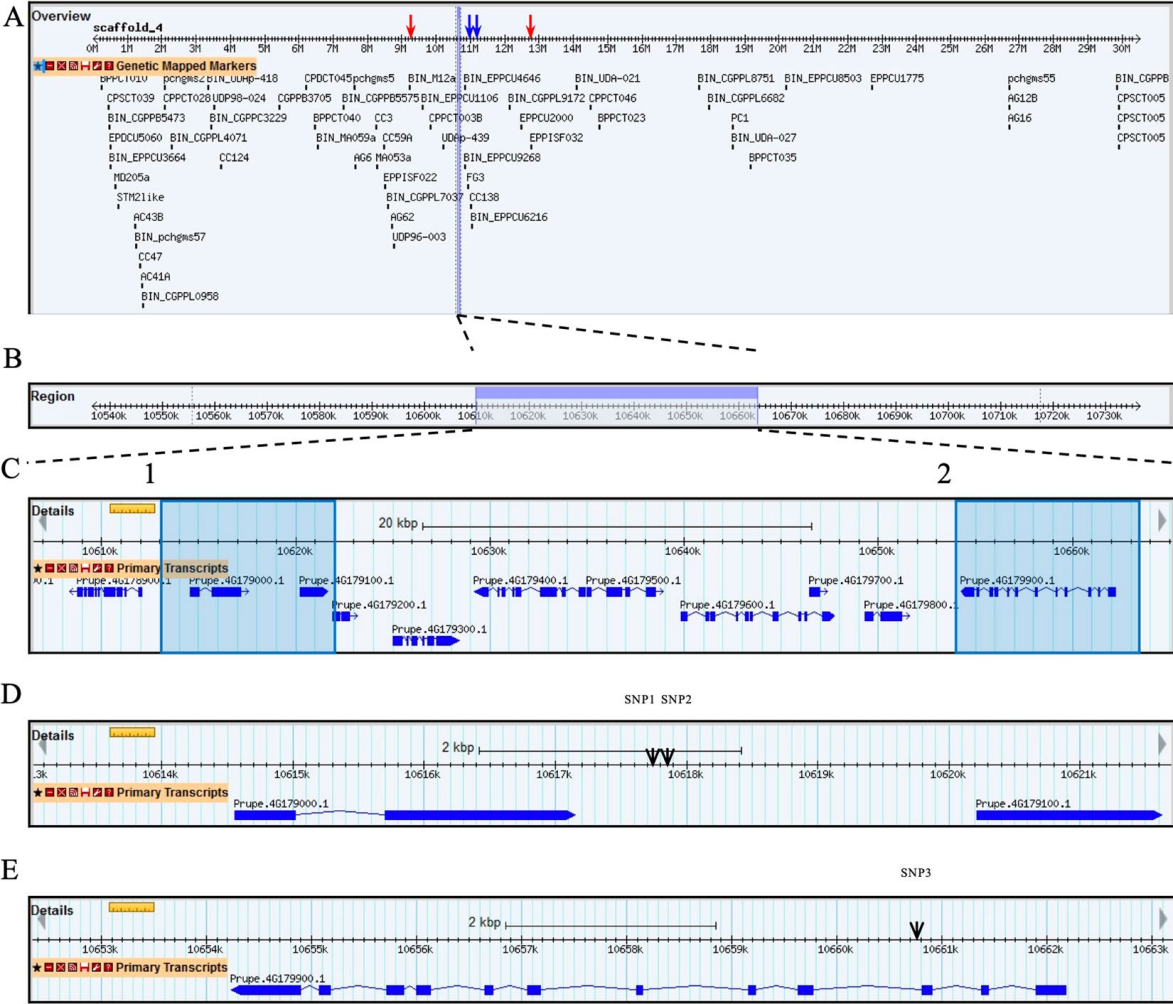
Graphical representation of linkage group 4 (LG4) and corresponding region of association to fruit development period (FDP) and maturity date (MD). (**A**) LG4 and the genetically mapped markers on LG4. The position of the original qMD4.1 locus (Eduardo *et al*., 2011) is marked by red arrows and the position of the refined qMD4.1 locus (Pirona *et al*., 2013) is marked by blue arrows. (**B**) General genomic region identified by the genome-wide association study (GWAS) for MD/FDP. (**C**) Detailed genomic region identified by GWAS for MD/FDP showing the genes found within this region and the two regions where single nucleotide polymorphisms (SNPs) were identified as indicated by the light blue sections numbered 1 and 2. (**D**) Detailed genomic region showing the location of SNP1 and SNP2 (corresponds to region 1 in **C**), (**E**) Detailed region showing the location of SNP3 (corresponds to region 2 in **C**).

Manhattan plots were generated for FDP and MD BLUPs using the mixed linear model (MLM) procedures (Figs 3 and 4). The MLM is considered a superior and more robust procedure compared to the general linear model (GLM) when conducting GWAS because it accounts for both population structure and relatedness^24,25^. Using this approach, seven SNPs were found to be associated with FDP at P < 0.05, but only three of these SNPs were associated with FDP at P < 0.01 (Table 3). Eight SNPs were found to be associated with MD at P < 0.05, however, only five of these SNPs were associated with MD at P < 0.01 (Table 3). The three SNPs identified at P < 0.01 for FDP were also observed for MD. These SNPs covered a region of 43,067 bp (chromosome 4: 10,617,717–10,660,784) (Fig. 5); we refer to this region as the MD/FDP locus. The SNPs associated with the MD/FDP locus were located at 10,617,717 bp (SNP1), 10,617,843 bp (SNP2) and 10,660,784 bp (SNP3), respectively (Fig. 5). MD/FDP SNPs 1 and 2 were located in intergenic regions and SNP 3 fell within an intron of the gene Prupe.4G179900 (Fig. 5).

**Table 3:** Single nucleotide polymorphisms (SNPs) identified using best linear unbiased predictors (BLUPs) for MD and FDP and their genomic and gene positions and orientations and the putative gene functions and gene orthologs for those genes that contained SNPs.

Trait	SNP Number	Locus	Genomic Position (bp)	Genomic Location	Gene^a^	Gene Ortholog^b^
FDP^^^,*/MD^^^,*	1	4	10617717	intergenic		
FDP^^^/MD^^^	2	4	10617843	intergenic		
FDP^^^,*/MD^^^,*	3	4	10660784	intragenic	Prupe.4G179900.1	AT5G53860.2
MD^^^^	4	4	12713584	intergenic		
FDP^^^^/MD^^^	5	4	12717141	intergenic		
FDP^^^^/MD^^^	6	4	12749548	intergenic		
FDP^^^^/MD^^^^	7	4	20843443	intergenic		
FDP^^^^/MD^^^^	8	4	20843475	intergenic		
FPD*	1	6	113793	intergenic		
FPD*	2	6	1399953	intergenic		

FDP, fruit development period; MD, maturity date.

*SNP is associated with the two most significant FDR values.

^^^SNP is significant at P < 0.01.

^^^^SNP is significant at P < 0.05.

^a^Putative gene functions are only listed for genes that contained SNPs identified by the GWAS.

^b^Gene orthologs are only listed for genes that contained SNPs identified by the GWAS.

The SNP with the strongest association to MD/FDP was SNP 3 (chromosome 4:10660784) (Table 3). Based on the GWAS of the BLUPs, SNP 3 had a *p*-value of 8.18E-09 and 4.64E-09 and an r^2^ of 0.31 and 0.36 for FDP and MD, respectively (Table 4). This locus accounted for 52% of the phenotypic variation of MD/FDP in our population (Table 5). We identified three haplotypes at this locus including TT, CT, and CC, which on average associated with early, mid and late maturing phenotypes, respectively (Table 5). Our results indicated that selection for TT alleles would have the potential to decrease average FDP by 14% (15.4 d) and MD by 7% (15.3 d). In contrast, if the objective is to breed for later maturing peaches, selection for CC alleles would have the potential to increase average FDP by 16% (18.4 d) and MD by 8% (18.2 d). Based on these results, selection for lateness would be slightly more effective than selection for earliness. This degree of asymmetry of selection response indicated that: (a) major gene effects are likely present in both MD and FDP traits; and (b) there may be more genes fixed or toward fixation for both lateness and long FDP than for earliness and short FDP.

**Table 4:** Association statistics of loci most significantly associated with the MD and FDP traits.

Trait	SNP^^^ Position	2012 Probability	r^2^	2013 Probability	r^2^	Combined Years Probability	(BLUPs) r^2^
FDP^^^^	chromosome 4 :10660784	6.86E-09**	0.31	1.61E-08**	0.31	8.18E-09**	0.30
MD^^^^	chromosome 4:10660784	4.78E-09**	0.36	2.28E-08**	0.30	4.64E-09**	0.36

**Marker trait association significant based on a 1% Benjamini-Hochberf FDR.

^^^The number of accessions for which the SNPs were called is 122 accessions.

^^^^Results reported based on mixed linear model (MLM).

**Table 5:** Variance statistics for allele classes of SNPs for loci most significantly associated with MD and FDP evaluated over two years in the study population.

Trait^^^	SNP^ Position	Probability	r^2^	Allele Class	Mean*	SD	Phenotype
FDP/MD	chromosome 4:10660784	<0.01	0.52/0.53	TT CT CC	97/213 109/225 131/246	7.76/7.14 14.60/14.18 12.70/12.05	Short FDP/Early Maturing Medium FDP/Mid Maturing Long FDP/Late Maturing

Means for MD and FDP are expressed as Julian days and days, respectively.

*Differences between allele class means are significant according to an F-test (α = 0.05).

^^^The number of accessions for which the SNPs were called is 122 accessions.

^^^Only traits in which we detected SNPs that were significant in at least one year and the means of the years combined are reported.

Based on a search in the reference genome (v2.0) we were able to locate 9 genes within the MD/FDP locus (Table 6). Three of these genes, namely Prupe.4G79900, Prupe.4G179800 and Prupe.4G179200 may play roles in the determination of MD/FDP in peach. Gene orthologs of Prupe.4G79900 in Arabidopsis and maize (*Zea maize*) are embryo defective 2737 (EMB2737)^26^ and embryo specific 12 (EMB12)^27^. These proteins belong to a family of EMB proteins that are required for normal embryo development in Arabidopsis and maize, respectively. It is adaptively essential that embryo development is complete prior to fruit attaining maturity in order to ensure that reproductively viable seeds are produced. Gene orthologs of Prupe.4G179800 in Arabidopsis and loblolly pine (*Pinus taeda*) are early nodulin-like protein 1 *(ENODL1)* and PtNIP1^28^, respectively. PtNIP1 is an ENODL transcript that is abundantly expressed in immature zygotic and somatic embryos of developing seeds, but is undetectable during later-stages of embryo development. Finally, gene orthologs of Prupe.4G179200 in Arabidopsis and rice (*Oryza sativa*) are purine permease 10 (PUP10) and OsPUP7^29^, respectively. A T-DNA insertion mutation in *OsPUP7* has been shown to delay flowering in rice. Interestingly, negative correlations between flowering times and FDP has been reported for peach^13,30^. This phenomenon may occur because a high correlation exists between temperature and FDP. Therefore, fruit of very early flowering genotypes may be slower to develop during stage I of fruit development compared to late flowering genotypes. This difference may be due to cooler temperatures following bloom experienced by early flowering genotypes^31–33^. Taken together, these results suggested that the determination of MD and FDP in peach may be regulated at the embryo level during seed and fruit development or during the transition between dormancy and bud break in spring. However, given the narrow flowering range observed in this study (data not shown), the determination of maturity period in our population is not likely strongly affected by bloom date as has been previously reported in other studies.

**Table 6:** Genes identified within the range of the SNPs significantly associated with maturity date/fruit development period (MD/FDP).

Gene	Transcript Start	Transcript End	Genomic Orientation	Putative Gene Function	Gene Ortholog
Prupe.4G179100.1	10620213	10621628	+	Polynucleotidyl transferase, ribonuclease H-like superfamily protein	AT2G36110.1
Prupe.4G179200.1	10621897	10622773	+	Purine permease 10	AT4G18210.1
Prupe.4G179300.1	10625033	10628440	+	Unknown	AT4G16807.1
Prupe.4G179400.1	10629172	10634877	−	Unknown	AT2G30700.1
Prupe.4G179500.1	10635017	10638518	+	Unknown	AT5G62270.2
Prupe.4G179600.1	10639863	10647757	+	Ypt/Rab-GAP domain of gyplp superfamily protein	AT2G30710.1
Prupe.4G179700.1	10646458	10646951	+	Unknown	Unknown
Prupe.4G179800.1	10649308	10651183	+	Early nodulin- like protein 1	AT5G53870.1
Prupe.4G179900.1	10654233	10662179	−	Embryo defective 2737	AT5G53860.2

**Figure 6:**
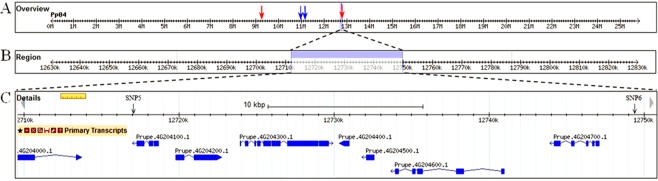
Graphical representation of linkage group 4 (LG4) and the corresponding region of association with the maturity date (MD) trait. (**A**) General overview of LG4. The position of the original qMD4.1 locus (Eduardo *et al*., 2011) is marked by red arrows and the position of the refined qMD4.1 locus (Pirona *et al*., 2013) is marked by blue arrows. (**B**) General region identified by the genome-wide association study (GWAS) for MD. (**C**) Detailed genomic region identified by the GWAS for MD and the genes and single nucleotide polymorphisms (SNPs) found within this region.

Two additional SNPs were identified for MD on LG 4 at P < 0.01, but not for FDP (Table 3). The additional SNPs associated with MD were located at 12,749,548 bp (SNP 4) and 12,717,141 bp (SNP 5) spanning a region of 32,407 bp (Fig. 6); we refer to this locus as MD. Both of these SNPs were located in intergenic regions. Based on a search in the reference genome (v2.0) we were able to locate seven genes within the MD locus (Fig. 6). Our MD locus fell within the qMD4.1 locus previously identified by Eduardo *et al*.^22^ (Fig. 6).

### The MD/FDP locus

There have been at least 10 other loci identified for MD/FDP on LG 4^17,18,21,22,34–36^. The positions of all of the reported loci that we were able to locate on the peach physical map co-located with or were in close proximity to our MD/FDP locus (Figs 5 and 6). Our MD/FDP locus fell within the qMD4.1 locus identified by Eduardo *et al*.^22^ and was nearby the refined qMD4.1 locus identified by Pirona *et al*.^18^. The qP-Fw4.1 locus reported by da Silva Ling *et al*.^36^ was positioned on chromosome 4: 10,665,019. This QTL is only 4,235 bp downstream from our reported MD/FDP locus. The BPPCT015 QTL reported by Song *et al*.^17^, was also positioned on chromosome 4: 12557824..12558026. This QTL is 1,897,040 bp upstream from our reported MD/FDP locus. Finally, our MD/FDP locus is 547,564 bp downstream qMD4 and qFDP, the recently reported QTLs for MD and FDP, respectively^21^. These results suggest that the MD/FDP locus reported here is the same as those MD/FDP loci reported in other studies.

Pirona *et al*.^17^ demonstrated that the MD trait is localized to a 220 kb region located on LG 4 of the peach genome. Of the 25 genes identified in this region, ppa008301m was a candidate for a major locus controlling MD in peach. In both populations studied by Pirona *et al*.^17^, ppa008301 had an in-frame insertion of 9 bp in the last exon that co-segregated with the MD locus. We identified three SNPs within gene ppa008301m; S4_11106857, S4_11106858, and S4_11106861 (Supplementary File 1). However, none of these SNPs showed significant association with MD/FDP. There are several reasons that may explain the discrepancies observed between our data and those reported by Pirona *et al*. The most evident explanation is that the population structure of the CxA/WxBy populations used by Pirona *et al*.^18^ are bi-parental mapping populations, and therefore, only contain the genes present in their parents. In contrast, our population consists of a wide range of germplasm containing much more genetic diversity compared to the mapping population used by Pirona *et al*. MD and FDP may be regulated by several genes that occur in all peach accessions and these genes may act redundantly in the peach genome as a whole. Therefore, it is conceivable that both qMD4.1 and the MD/FDP locus contain different genes that regulate MD/FDP and these genes or genomic regions are co-located. In this situation ppa008301 may be responsible for regulating MD/FDP in the CxA/WxBy populations, while a separate gene may be regulating MD/FDP in our population.

## Conclusion

In conclusion, using an association mapping population consisting of a uniquely variable assortment of peach accessions, we identified many SNPs and genomic regions that associated with MD and FDP. We were also able to substantiate the use of GBS and GWAS in the efficient and accurate identification of molecular markers that associate with maturity in peach. The data reported here will aid peach and other *Prunus* spp. breeders in MASS targeted towards extending the peach production season. The adoption of MASS in peach breeding has been slow, but much success is anticipated in future years. In several Rosaceae fruit tree crops, successful MASS applications have been reported, substantiating the feasibility and value of conducting MASS in peach. The recent availability of the peach physical map will continue to allow for marker saturation around important traits, and therefore, it is foreseeable that marker assisted breeding (MAB) in peach will prove successful over the years to come. The findings reported here will also allow for a transferable enhanced understanding of the genetic regions, components and mechanisms underlying fruit maturity in peach.

## Materials and Methods

### Plant material and phenotyping

To ensure adequate coverage of the peach genome, an association mapping population collected from a wide range of localities across North America was assessed. Hundred thirty-two peach genotypes were used in this study^3^ (Table 1). All of the trees are located in a single farm at the University of Guelph’s experimental plots in Vineland. The accessions were originally obtained from various localities across North America or were generated and maintained. Each accession was grafted on a Bailey rootstock. The trees were planted side by side with a spacing of 4.5 m within and 5.5 m between rows and trained using the open center technique. Rows were oriented in a north/south direction and trees ranged in age from 5–10 years. Four trees from each accession were planted adjacent to one another in a single row or two trees from one accession in one row and the other two trees from the same accession in the adjacent row. Pruning was performed yearly and standard cultural practices were applied. The fruit were thinned before pit hardening to a load of 200–300 fruit per tree when possible. This thinning strategy ensured that fruit size was not limited by competition and that the fruit load was representative of normal farming practices. Three trees from each accession were manually evaluated for BD, MD and FDP over 2 years, which corresponded to the 2012 and 2013 growing seasons.

20–30 fruits from each tree were manually evaluated in the field for color change as well as fruit size and firmness in order to determine the proportion of fruit on each tree that was at the market mature stage. In order to ensure adequate phenotypic evaluation of MD/FDP, ripening dates were recorded at least twice a week during the early and late ripening season, and at least three times a week in the middle of the ripening season. The phenotypes for BD were measured as the number of Julian days from January 1^st^ to the date of 50% bloom (Julian day when ~50% of the flowers on three trees from each genotype reached the full bloom stage). The phenotypes for MD were measured as the number of Julian days from January 1^st^ to the date of ripening (Julian day when ~50% of the fruit on three trees from each genotype had reached the market mature stage). The interval between BD and MD was considered as FDP (days).

### DNA extraction and genotyping

DNA extraction from young leaves was carried out using the Norgen plant and fungus DNA extraction kit (Norgen, 3430 Schmon Parkway Thorold, Ontario Canada), with some modifications. These modifications include (1) using 30 mg of lyophilized tissue instead of 50 mg of fresh plant tissue for the extraction (2) increasing the lysis solution to 250 µL and the lysis additive to 50 µl, (3) soaking the lyophilized tissue in lysis solution and additive for 20 minutes instead of 5 minutes at room temperature and manually mixing the solution by shaking every five minutes (4) samples were incubated for 30 minutes instead of 10 minutes and manually shaken every 10 minutes; Increasing the soaking and incubation time served the purpose of increasing the quantity of DNA extracted, (5) binding solution was heated in the microwave for 12 seconds on high and manually mixed by shaking, (6) drained columns were allowed to sit in the fume hood for 5 minutes to ensure residual ethanol evaporates (7) elution buffer was heated in microwave for 12 seconds on high and manually mixed by shaking. After the column-based extraction, DNA was qualified using gel electrophoresis and assessed for quantity using the NanoDrop ND-1000 spectrophotometer.

### Genotyping by sequencing

To determine the target region(s) that contain the candidate gene(s) responsible for regulating MD/FDP, we used a genotyping by sequencing (GBS) approach. This approach involved genotyping each accession in our population and taking accurate phenotypic MD/FDP data. This data was then used to identify polymorphisms associated with MD/FDP using the Tassel 5.0 GBS pipeline. To obtain the genotypic data, DNA was collected from each accession as described previously. The DNA was prepared into GBS libraries and sequenced on four lanes of Illumina HiSeq (Illumina, 5200 Illumina Way, University City, San Diego, CA) sequencing using the GBS protocol at the Institute for Genomic Diversity (IGD) at Cornell University, USA. The type II restriction endonuclease A*pe*KI was used as the adapter for the protocol. The genotypic data were processed using the default parameters^37^ of the TASSEL-GBS pipeline (version 5.0, Institute for Genomic Diversity, Ithaca, NY)^38^. SNPs were filtered for a minor allele frequency of 2% and sites were filtered for a minimum site coverage of 0.8 and a minimum taxon coverage of 0.5^39^. The Burrow Wheeler Aligner (BWA) (version 0.6.2)^40^ was the read mapping software used for the analysis. The *Prunus persica* v 2.0 genome assembly from www.rosaceae.org was used to align the GBS data and tag the SNPs. The GBS data were deposited on the NCBI SRA database and can be accessed under the BioProject identifier PRJNA506576.

The R package software ‘GAPIT’ was used for the association analysis to determine the regions of the peach genome which associated with MD/FDP. The phenotypic and genotypic data were used to conduct association analysis (Tables S3, S4). Because we used BLUPs to model the genotypic effect as a random effect, the model accounted for variance due to genotype × year (i.e. G × E). For this reason, the QTLs identified by the BLUPs were only illustrated. However, SNP detection over multiple years has been included as supplemental figures for comparison (Figs 2 and S1). We only considered SNPs that were significant in at least one year and the means of the years combined.

The processed genotypic data was then converted into BLUPs and used with the phenotypic data in a genome-wide association analysis to identify SNP polymorphisms associated with the evaluated traits. The genome-wide association analysis was performed with the compressed mixed linear model^25^ implemented in the GAPIT R package^41^ and with the general linear model (GLM) and mixed linear model (MLM) implemented in the TASSEL software (version 5.0 Institute for Genomic Diversity, Ithaca, NY). Evaluation of incorporating different amounts of missing data (5–20% missing data) was performed, but rejected because it made no difference to the associations found. Each accession was sequenced twice.

### Association mapping analysis

The Tassel 5.1 GBS pipeline was used to identify polymorphisms and the R package software ‘GAPIT’ (http://www.maizegenetics.net/gapit) was used for the association analysis.

### Statistical analyses

The genotype, year, genotype x year and residual effects for MD and FDP were estimated by using best linear unbiased predictors (BLUPs)^42^. The BLUPs were also used to generate estimated breeding values (EBVs), which were used to conduct genotypic correlation analysis and for the GWAS in TASSEL. The BLUP analysis was conducted using the lme4 package in R^43^. Genotype, year and their interactions were treated as random effects. For MD and FDP the model genotype, year and genotype x year was used. The variance components were then used to estimate broad sense heritability (H^2^) for both traits. Heritability was estimated by using the formula H^2^ = [genotypic variance/(variances of the interaction effects + variance of the residual)].

Pearson phenotypic and genotypic correlations with significance levels for MD and FDP were conducted using the Hmisc package in R^44^. Both year × year and trait × trait correlation matrices were developed for the phenotypic results. For the year × year correlations, means within years were used to generate correlations across years for each individual trait. For trait by trait correlations means for all years combined were used to generate the correlation matrix for individual traits. A trait × trait correlation matrix was generated for the genotypic data using the EBVs generated from the BLUPs.

A principal component analysis (PCA) was conducted in the software PLINK (version 1.9) using the hap maps generated from the GBS data. The PCA was conducted in order to account for genetic relationships within the study population. An r^2^ threshold of 0.02 was used to conduct the PCA and 100 polymorphic positions were assigned for the analysis. The eigenvalues generated from the PCA were used to generate a scree plot. The number of PCs that had eigenvalues above 1.00 was used for the GWAS in TASSEL to account for relatedness.

In order to visualize the structure and relatedness of the population, a multidimensional scaling (MDS) plot was generated using the scatterplot3d package in R^45^. The plot was generated using the first three principal components (PCs) generated from our PCA (Fig. S3).

A linkage disequilibrium (LD) decay analysis was conducted for individual chromosomes of the population (Table S2). The purpose of the LD decay analysis was to determine whether the number of SNPs identified from the GBS sequencing sufficiently covered the genome. The LD decay analysis was conducted using PLINK (version 1.9). An r-square cutoff of 0.2 was chosen to define the extent of LD in the population. All marker pairs were compared for LD calculations and markers within the range of the total number of nucleotides within each chromosome were compared for LD calculations. LD decay plots for each chromosome were then generated using the ggplot2 package in R (Fabio Marroni’s Blog, 2011, https://fabiomarroni.wordpress.com/) to visualize the data. In order to account for experimental error in the GWAS in TASSEL, a Benjamini-Hochberg false discovery rate (BH-FDR) correction was conducted. BH-FDR corrections were determined using R (version 3.2.3). The BH-FDR correction data was used to adjust p-values and assign significance thresholds to identify SNPs that associated with MD and FDP. Significance thresholds for SNP discovery were identified using a type 1-error rate of P < 0.05 and P < 0.01.

## Supplementary information

Supplementary Information
