# Supplementary material for: Refining the Genomic Region Containing a Major Locus Controlling Fruit Maturity in Peach
Source: Sci Rep. 2019 May 17;9:7522. doi: 10.1038/s41598-019-44042-4 (PMC6525192; doi:10.1038/s41598-019-44042-4)
Supplement: Supplementary file 1 — Supplementary Information [file 41598_2019_44042_MOESM1_ESM.pdf]

Refining the Genomic Region Containing a Major Locus Controlling Fruit Maturity in Peach

Elsadr H.[1], Sherif S.[2], Banks T.[3], Somers D.[3], and Jayasankar S.[1]

[1]University of Guelph, Department of Plant Agriculture, Ontario, Canada

[2]Alson H. Smith Jr. Agricultural Research and Extension Center, Virginia Tech, Winchester, Virginia, USA

[3]Vineland Research and Innovation Centre, Department of Plant Agriculture, University of Guelph, Guelph, Ontario, Canada

.

Corresponding author: Jayasankar Subramanian

Address: University of Guelph, Department of Plant Agriculture.

4890 Victoria Av. N., P.O. Box 7000 Vineland Station, ON, L0R 2E0 Canada

Telephone: +1 (905) 562-4141 x134

Fax: +1 (905) 562-3413

E-mail: jsubrama@uoguelph.ca

Supplementary Data

Table S1: Peach genotypes used in this study and their respective pedigrees

Accession	Pedigree	Accession	Pedigree
Allstar	Fayette x Newhaven	V99131	Harson x o.p.
Blazingstar	Fayette x Newhaven	V99141	Harrow Fair x Emilia
Bounty	[(Halberta x o.p.) x Redskin] x (Loring x [(Hiley x Fireglow) x Fireglow) x Fireglow])	V99161	Harrow Fair x KV930455
Coralstar	Fayette x Newhaven	V99171	Harrow Fair x o.p.
Desiree	Garnet Beauty x Sentry	V99221	Jerseyglo x Emilia
Garnet Beauty	Redhaven mutation	V99231	Jerseyglo x HW268
Gloria	NJ304 x J19-19-862144	V99232	Jerseyglo x HW268
Glowing Star	Fayette x Newhaven	V99241	Jerseyglo x o.p.
H.Beauty	Cresthaven x Harken	V99242	Jerseyglo x o.p.
H.Diamond	Redskin x Harbinger	V99261	HW268 x KV930455
Harson	Redskin x Sunhaven	V99262	HW268 x KV930455
HW261	Cresthaven x H4219	V99263	HW268 x KV930455
HW268	HW206 x o.p.	V993110	Vinegold x V92131
HW269	Cresthaven x SH-450	V99316	Vinegold x V92131
HW271	Vollie x HW224	V99321	Vinegold x V84101
HW272	HW225 x H6744005	V99322	Vinegold x V84101
HW273	Biscoe x SH-415	V99323	Vinegold x V84101
HW274	Redskin x Harrow Diamond	V99325	Vinegold x V84101
HW275	H243 x Harrow Diamond	V99326	Vinegold x V84101
HW276		V99351	HW271 x V88272
KV930455 (Crimson Rocket)	KV881465 x MA6-1-90	V99352	HW271 x V88272
Messina	D90-9 x NJ318	V99371	V88272 x HW271
NJF15	B7-6-151-752080 x NJF7	V99372	V88272 x HW271
NJF16	A43-143-782081 x NJF4	V99381	V88272 x White Lady
NJF18	H15-20-90258 x A34-160-782118	V99382	V88272 x White Lady
Redstar	Newhaven x o.p.	V99383	V88272 x White Lady
Risingstar	Newhaven x Jim Dandee	V99384	V88272 x White Lady
Starfire	Fayette x Newhaven	V99385	V88272 x White Lady
Sweet-N-Up	KV882304 x BO87021003	V99391	V88272 x o.p.
V200072	V95013 x Catherina	V99392	V88272 x o.p.
V200114	V950125 x Catherina	V99393	V88272 x o.p.
V200161	HW 261 x V55061	V99394	V88272 x o.p.
V200171	HW 261 x O.P.	V99395	V88272 x O.P.
V200172	HW 261 x O.P.	V99396	V88272 x O.P.
V203071	Venture x KV930278	V99401	Flamina x V85384
V85221	Harrow Diamond x V68101	V99402	Flamina x V85384
V85331	Newhaven x Vivid	V99403	Flamina x V85384
V85384	Harrow Beauty x V75012	V99404	Flamina x V85384
V853914	Harrow Beauty x V792502	V99405	Flamina x V85384
V88021	Veeglo x Harrow Diamond	V99406	Flamina x V85384
V88063	Veeglow x V39071	V99407	Flamina x V85384
V88261	Redhaven x Brighton	V99408	Flamina x V85384
V88262	Redhaven x Brighton	V99411	Flamina x HW268
V88272	PES Redhaven x Sentry	V99412	Flamina x HW268
V92131	V68051 x V790737	V99413	Flamina x HW268
V92301	Harrow Diamond x V790638	V99414	Flamina x HW268
V94021	Harson x o.p.	V99415	Flamina x HW268
V94091	Newhaven x V88063	V99422	Flamina x o.p.
V95191	V790638 x Harblaze	V99423	Flamina x o.p.
V95192	V790638 x Harblaze	V99431	Brighton x HW271
V95241	Yumyeong x o.p.	V99432	Brighton x HW271
V95275	V88272 x Yumyeong	V99471	Cresthaven x V85384
V95276	V88272 x Yumyeong	V99481	Cresthaven x Emilia
V95277	V88272 x Yumyeong	V99482	Cresthaven x Emilia
V95281	V88272 x Eden	V99483	Cresthaven x Emilia
V96151	Newhaven x HW106	V99491	Cresthaven x HW268
V97171	Garnet Beauty x Yumyeong	V99492	Cresthaven x HW268
V97211	Sentry x H. Diamond	V99501	Cresthaven x HW271
V97331	V92262 x o.p.	V99502	Cresthaven x HW271
V97381	Andross x o.p.	V99503	Cresthaven x HW271
V98221	Harrow Fair x V85384	V99504	Cresthaven x HW271
V98321	H9301117 x open	V99511	Cresthaven x o.p.
V98331	H9301168 x o.p.	V99512	Cresthaven x o.p.
V99043	Harflame x o.p.	V99513	Cresthaven x o.p.
V99081	H. Diamond x SB-17	Vivid	Sunhigh x (Early Halehaven x Envoy)
V99121	Harson x KV930455	Vollie	Redskin x Kalhaven

Table S2: Number of SNPs required to sufficiently detect recombination on each chromosome in the study population

Chromosome	SNPs Detected from GBS sequencing	Bases at LD Decay	Bases in Genome*	Markers Needed for Association**
**1**	3368	147750	47800000	324
**2**	2569	282145	29800000	106
**3**	1647	166007	22100000	133
**4**	2817	409919	30400000	74
**5**	1646	277659	18400000	66
**6**	2894	650608	28800000	44
**7**	1705	99146	22700000	229
**8**	1733	91382	21700000	237
**Average**	2297	265577	27712500	152

*Values taken from Gbrowse (Prunus persica version 2.0.a1 assembly) using the Genome Database for Rosaceae (GDR)

**Values obtained by dividing the number of bases in the genome by the number of bases at LD Decay

Table S3: Fruit maturity dates (MD) for the 132 peach accessions used in the GWAS study.

Lines	MD 2012	MD 2013	MD Average
Allstar	234	235	235
Blazingstar	223	231	227
Bounty	226	239	233
Corelstar	231	236	234
Desiree	200	217	209
G. Beauty	205	216	211
Gloria	231	242	237
Glowing Star	242	244	243
H.Beauty	235	240	238
H.Diamond	194	206	200
Harson	214	228	221
HW261	217	226	222
HW268	239	243	241
HW269	199	204	202
HW271	216	230	223
HW272	220	225	223
HW273	236	241	239
HW274	199	215	207
HW275	198	210	204

HW276	201	212	207
KV930455	227	232	230
Messina	238	247	243
NJF15	210	224	217
NJF16	215	224	220
NJF18	208	222	215
Redstar	223	228	226
Risingstar	207	216	212
Starfire	224	231	228
Sweet-N-Up	238	243	241
V200072	261	268	265
V200114	263	273	268
V200161	212	228	220
V200171	210	224	217
V200172	235	239	237
V203071	229	238	234
V85221	193	202	198
V85331	220	227	224
V85384	236	242	239
V853914	208	222	215
V88021	192	201	197
V88063	233	234	234
V88261	207	215	211
V88262	220	229	225
V88272	207	220	214
V92131	222	233	228
V92301	200	210	205
V94021	241	244	243
V94091	208	223	216
V95191	203	219	211
V95192	NA	227	227
V95241	235	243	239
V95275	213	226	220
V95276	226	235	231
V95277	221	232	227
V95281	228	237	233
V96151	218	225	222
V97171	233	236	235
V97211	190	198	194
V97331	200	214	207
V97381	246	250	248
V98221	218	227	223
V98321	NA	216	216
V98331	220	231	226

V99043	214	225	220
V99081	205	221	213
V99121	216	224	220
V99131	216	231	224
V99141	242	248	245
V99161	227	234	231
V99171	202	217	210
V99221	NA	252	252
V99231	240	245	243
V99232	242	250	246
V99241	241	245	243
V99242	238	239	239
V99261	221	232	227
V99262	226	235	231
V99263	244	253	249
V993110	216	226	221
V99316	217	226	222
V99321	212	224	218
V99322	206	219	213
V99323	211	222	217
V99325	207	221	214
V99326	208	220	214
V99351	207	217	212
V99352	204	217	211
V99371	200	220	210
V99372	201	215	208
V99381	204	215	210
V99382	219	NA	219
V99383	207	218	213
V99384	212	220	216
V99385	207	218	213
V99391	191	201	196
V99392	205	219	212
V99393	203	218	211
V99394	202	219	211
V99395	201	217	209
V99396	202	216	209
V99401	263	269	266
V99402	255	271	263
V99403	262	272	267
V99404	251	262	257
V99405	261	270	266
V99406	NA	270	270
V99407	NA	272	272

V99408	NA	272	272
V99411	252	258	255
V99412	244	247	246
V99413	243	253	248
V99414	245	261	253
V99415	256	267	262
V99422	255	262	259
V99423	207	221	214
V99431	201	216	209
V99432	206	217	212
V99471	244	250	247
V99481	253	261	257
V99482	221	235	228
V99483	232	234	233
V99491	246	252	249
V99492	243	245	244
V99501	215	226	221
V99502	245	251	248
V99503	213	226	220
V99504	242	246	244
V99511	247	250	249
V99512	243	247	245
V99513	243	250	247
Vivid	221	233	227
Vollie	227	240	234

Table S4: Fruit developing periods (FDP) for the 132 peach accessions used in the GWAS study.

Lines	FMD 2012	FMD 2013	FMD Average
Allstar	126	111	119
Blazingstar	115	107	111
Bounty	120	116	118
Corelstar	126	113	120
Desiree	93	92	93
G. Beauty	97	92	95
Gloria	120	114	117
Glowing Star	132	119	126
H.Beauty	127	115	121
H.Diamond	86	80	83
Harson	106	103	105
HW261	107	99	103

HW268	131	118	125
HW269	90	79	85
HW271	108	104	106
HW272	111	99	105
HW273	128	117	123
HW274	92	90	91
HW275	88	83	86
HW276	94	88	91
KV930455	122	109	116
Messina	133	124	129
NJF15	103	99	101
NJF16	108	100	104
NJF18	101	97	99
Redstar	112	102	107
Risingstar	98	89	94
Starfire	116	107	112
Sweet-N-Up	132	121	127
V200072	156	145	151
V200114	158	150	154
V200161	106	102	104
V200171	102	96	99
V200172	125	110	118
V203071	121	113	117
V85221	85	77	81
V85331	109	103	106
V85384	129	118	124
V853914	101	98	100
V88021	84	76	80
V88063	123	109	116
V88261	99	91	95
V88262	110	103	107
V88272	99	95	97
V92131	114	108	111
V92301	93	85	89
V94021	135	120	128
V94091	99	98	99
V95191	97	95	96
V95192	NA	104	104
V95241	127	116	122
V95275	106	101	104
V95276	120	110	115
V95277	113	106	110
V95281	121	113	117
V96151	111	101	106

V97171	125	111	118
V97211	82	74	78
V97331	94	89	92
V97381	137	126	132
V98221	111	102	107
V98321	NA	92	92
V98331	114	107	111
V99043	107	99	103
V99081	99	98	99
V99121	108	98	103
V99131	110	106	108
V99141	135	124	130
V99161	119	109	114
V99171	96	94	95
V99221	NA	129	129
V99231	132	119	126
V99232	137	128	133
V99241	134	123	129
V99242	130	114	122
V99261	114	107	111
V99262	119	111	115
V99263	136	127	132
V993110	108	100	104
V99316	109	101	105
V99321	103	96	100
V99322	99	94	97
V99323	103	96	100
V99325	98	96	97
V99326	101	96	99
V99351	101	93	97
V99352	98	93	96
V99371	93	92	93
V99372	94	90	92
V99381	97	91	94
V99382	112	NA	112
V99383	100	94	97
V99384	105	96	101
V99385	100	93	97
V99391	84	75	80
V99392	98	95	97
V99393	95	93	94
V99394	95	94	95
V99395	93	92	93
V99396	94	91	93

V99401	158	145	152
V99402	150	147	149
V99403	157	149	153
V99404	145	138	142
V99405	155	147	151
V99406	NA	146	146
V99407	NA	149	149
V99408	NA	149	149
V99411	146	136	141
V99412	138	123	131
V99413	138	129	134
V99414	137	133	135
V99415	148	141	145
V99422	149	139	144
V99423	101	97	99
V99431	94	91	93
V99432	97	89	93
V99471	138	127	133
V99481	146	137	142
V99482	113	109	111
V99483	126	111	119
V99491	138	128	133
V99492	135	120	128
V99501	108	101	105
V99502	137	127	132
V99503	107	102	105
V99504	134	120	127
V99511	138	126	132
V99512	NA	119	119
V99513	134	124	129
Vivid	114	109	112
Vollie	119	115	117

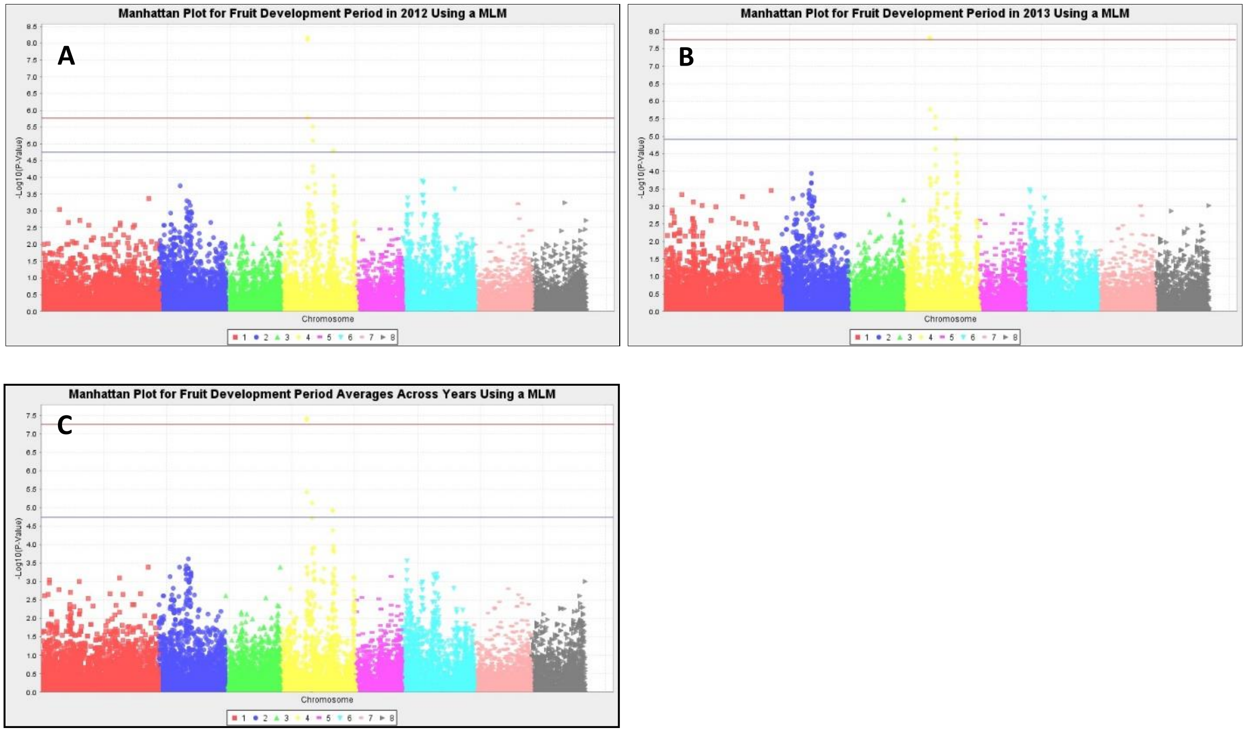

Fig S1. Manhattan plots for fruit development period best linear unbiased predictors (BLUPs) (A) in 2012 using a MLM, (B) in 2013 using a MLM and (C) averages across years using a MLM. Red and blue lines represent significance thresholds at $P < 0.01$ and $P < 0.05$, respectively using a Benjamini-Hochberg false discovery rate adjustment.

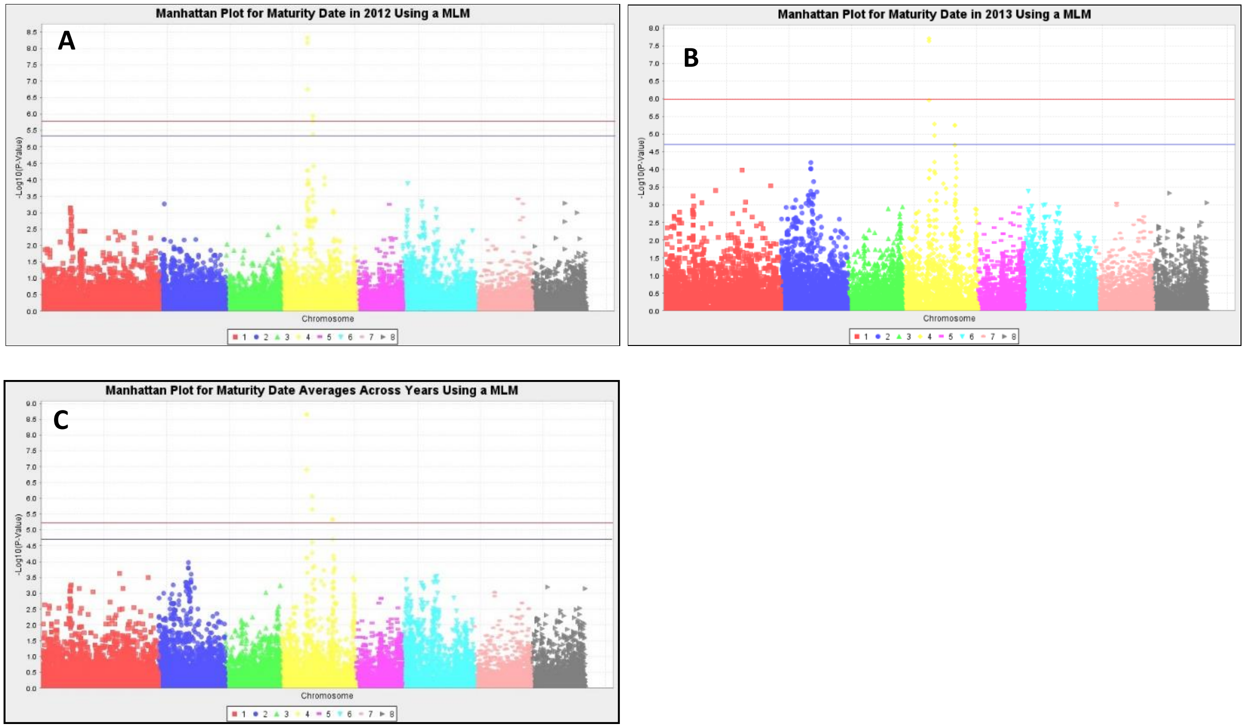

Fig S2. Manhattan plots for maturity date best linear unbiased predictors (BLUPs) (A) in 2012 using a MLM, (B) in 2013 using a MLM and (C) averages across years using a MLM. Red and blue lines represent significance thresholds at $P < 0.01$ and $P < 0.05$, respectively using a Benjamini-Hochberg false discovery rate adjustment.

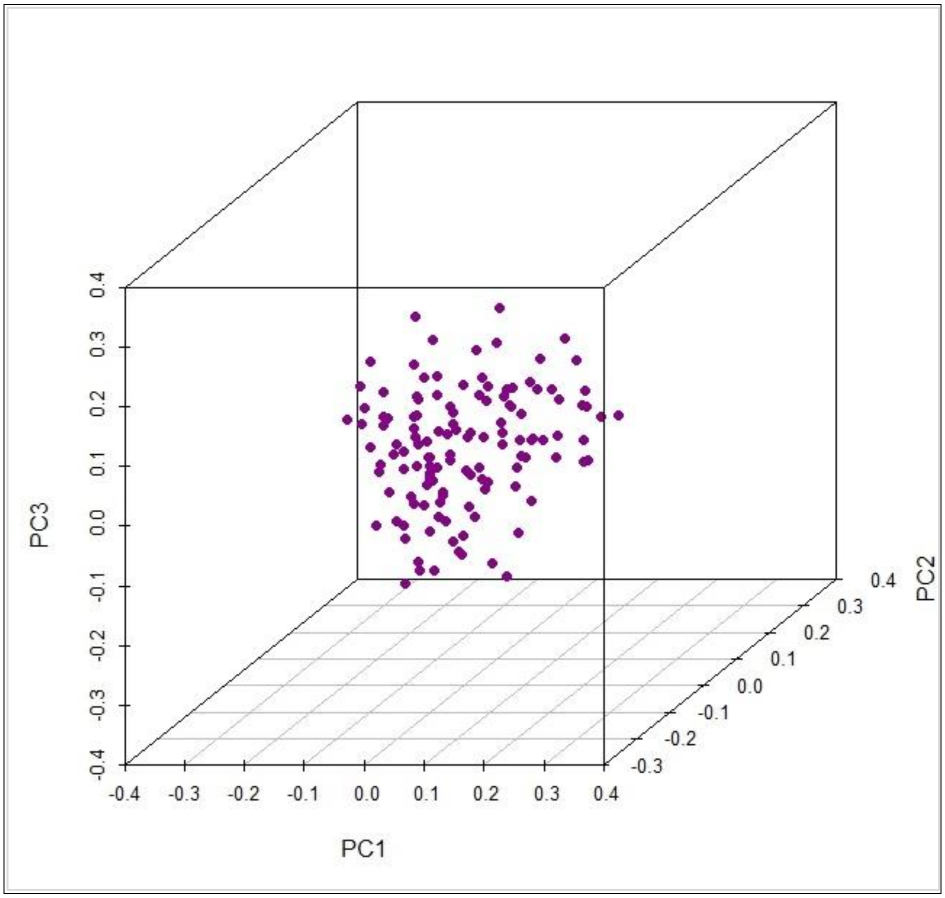

Fig S3. Multi dimensional scaling plot of the study population. Principle components were generated using the HapMap file produced from the GBS sequencing data. PC1-3, principle components 1-3.
